# MEMS development focusing on collaboration using common facilities: a retrospective view and future directions

**DOI:** 10.1038/s41378-021-00290-x

**Published:** 2021-08-12

**Authors:** Masayoshi Esashi

**Affiliations:** 1 MEMS CORE Co. Ltd. (CTO), Sendai, 981-3206 Japan; 2 Micro System Integration Center (μSIC) (senior research fellow), Tohoku University, Sendai, 980-0845 Japan

**Keywords:** Electrical and electronic engineering, Sensors

## Abstract

I have been developing MEMS (microelectromechanical systems) technology and supporting the industry through collaboration. A facility was built in house on a 20 mm square wafer for use in prototyping MEMS and ICs (integrated circuits). The constructed MEMS devices include commercialized integrated capacitive pressure sensors, electrostatically levitated rotational gyroscopes, and two-axis optical scanners. Heterogeneous integration, which is a MEMS on an LSI (large-scale integration), was developed for sophisticated systems using LSI made in a foundry. This technology was applied for tactile sensor networks for safe robots, multi FBAR filters on LSI, active-matrix multielectron emitter arrays, and so on. The facility used to produce MEMS on 4- and 6-inch wafers was developed based on an old semiconductor factory and has been used as an open hands-on access facility by many companies. Future directions of MEMS research are discussed.

## Introduction

MEMS (microelectromechanical systems) technology enables the fabrication of many small and value-adding devices such as sensors on wafers. Cheap devices can be produced in large volumes, but expensive semiconductor facilities and versatile knowledge are required.

**Fig. 1:**
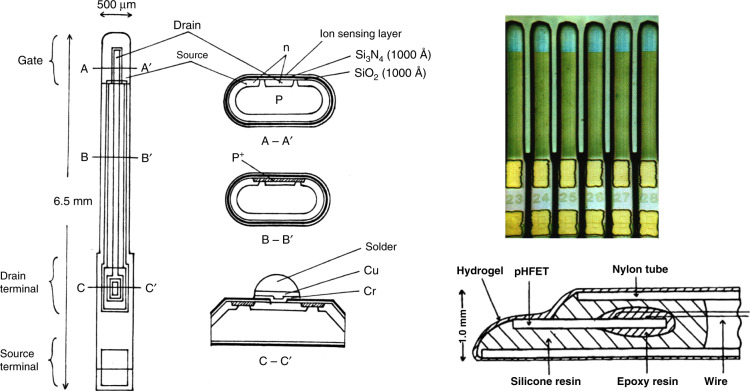
ISFET (ion-sensitive field-effect transistor)^1^.

I have been engaged in MEMS development and industrialization at Tohoku University for nearly half a century, starting when I was a student. Figure 1 shows an ISFET (ion-sensitive field-effect transistor), which was developed when I was a postgraduate student (1971–1975)^1^. My supervisor Prof. Tadayuki Matsuo was working at Stanford University, where MEMS technology was pioneered in the 1970s, and he informed me about MEMS technology. The gate insulator of an insulated gate FET is exposed to an electrolyte, and we can detect ions such as H^+^ in the electrolyte. Si probes with ISFETs were fabricated by MEMS technology to be encapsulated at the end of 1 mm outer diameter catheter tubes^2^. I have been focusing on MEMS packaging because it plays important role in the practical use of many MEMS devices. The ISFET catheter was commercialized in 1980 by Nihon Kohden Ltd. for the diagnosis of gastroesophageal reflux disease. The ISFET was also used for portable pH meters by ISFETCOM Co. Ltd.

**Fig. 2:**
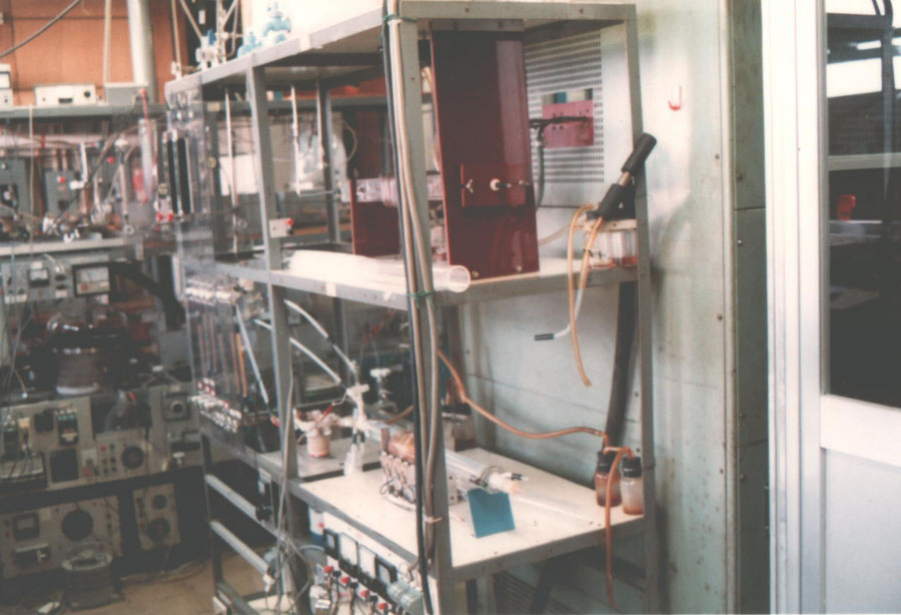
Prototyping facility for MEMS and ICs on 20 mm square wafers.

I made the MEMS prototyping facility that could process a 20 mm square wafer for an ISFET when I was a postgraduate student. I learned how to make the facility in the laboratory of Prof. Jun-ichi Nishizawa, who was a pioneer of semiconductor technology in Japan. The facility shown in Fig. 2 has been in use for initial stage prototyping, as will be described later.

When I was a research assistant from 1976 to 1980, I worked with students. We developed biomedical sensors such as catheter pressure sensors used in blood vessels.

In 1980, I became an associate professor and started to fabricate custom CMOS IC using a facility made in-house. I wrote a graphic editor program for layout, which was a good opportunity for me to learn to program. I made an LSI tester by connecting a circuit to the parallel I/O of a minicomputer made in DEC, which was also a good opportunity for me to learn about digital circuits. I wrote a textbook named “Fundamentals of integrated circuit design” (1986) (Baifukan) (in Japanese).

I have worked as a professor since 1990 and developed basic MEMS technologies as described below. MEMS devices were commercialized in collaboration with engineers dispatched from the industry. We developed heterogeneous integration by stacking MEMS on LSI, as will be described later. Since 2019, I have been working as a CTO of MEMS CORE Co. Ltd. and as a senior research fellow at the Micro System Integration Center (μSIC) at Tohoku University.

## Development of basic MEMS technologies

**Fig. 3:**
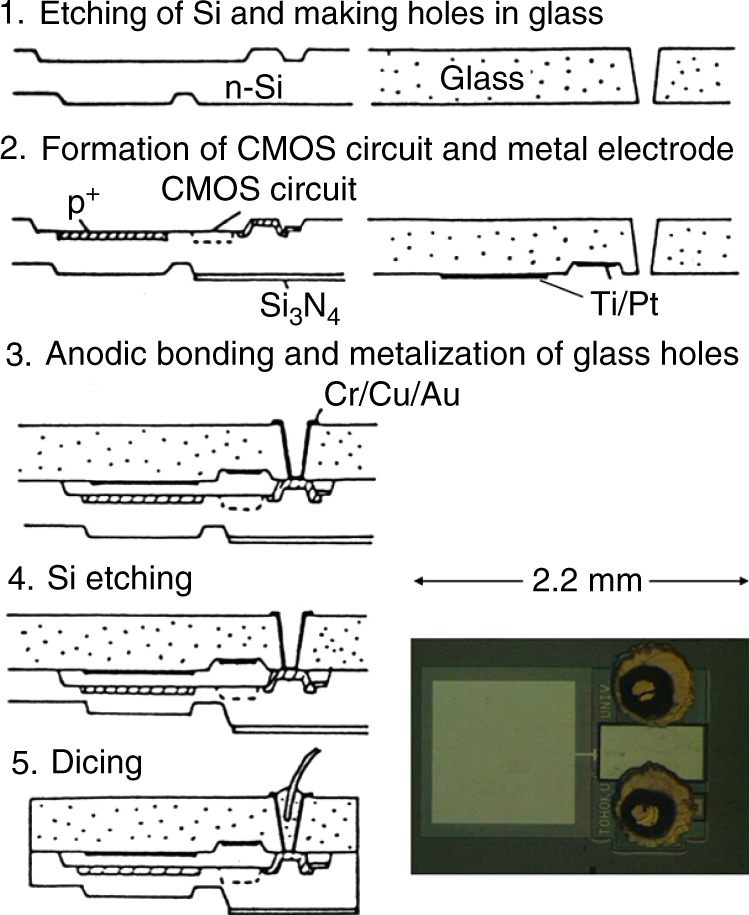
Fabrication process of the integrated capacitive pressure sensor^3^.

Capacitive sensors have the advantages of low power consumption; however, the circuit to detect capacitance has to be located close to the sensor and not be influenced by stray capacitances. Figure 3 shows the fabrication process of an integrated capacitive pressure sensor^3^. The capacitance detection circuit is fabricated on a Si wafer (2 in the figure). The wafer is anodically bonded to a glass wafer by applying −400 V to the glass at 400 °C (3 in the figure). Diaphragms are made by etching the Si wafer from the back side (4 in the figure), and the wafer is diced into chips (5 in the figure). This process is based on wafer-level packaging by which packaged small chips are obtained and the fabrication cost is reduced^4^. This sensor was commercialized by Toyoda Machine Works Ltd. (now JTECT Ltd.) as highly sensitive pressure sensors to monitor the clogging of filters in air conditioners^5^. The wafer-level, packaged MEMS devices were commercialized as capacitive vacuum sensors by Canon Anelva Corp^6^., MEMS switches for LSI testers by Advantest Corp^7^., and other MEMS devices.

**Fig. 4:**
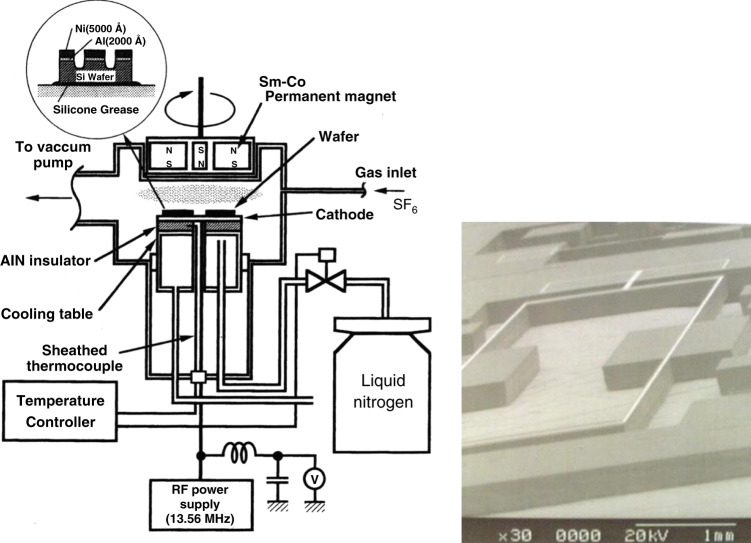
Deep reactive ion etching system^8^ and Si resonating gyroscope chip made by DRIE^9^.

The process equipment required was developed in our laboratory. Figure 4 shows a DRIE (deep reactive ion etching) system to make deep grooves and holes in a Si wafer^8^. A wafer etched through the thickness for a resonating gyroscope is also shown in Fig. 4^9^. The wafer has to be cooled down in this system, and hence, this is not as convenient as the DRIE system commercialized by Robert Bosch later, but we could fabricate various MEMS devices using our DRIE system.

### Open collaboration with industry

Approximately 130 companies have dispatched their employees to our laboratory an average of 2 years each. Some examples of commercialization based on our collaboration are described below.

**Fig. 5:**
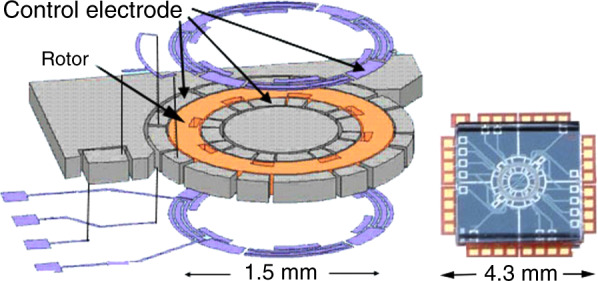
Electrostatically levitated rotational gyroscope^10^.

Figure 5 is an electrostatically levitated rotational gyroscope^10^. A ring Si rotor with an outer diameter of 1.5 mm rotates at 74,000 RPM, two axis rotations and three axis accelerations are measured simultaneously with high precision. High-speed digital signal processing by capacitive sensing and electrostatic actuation in all directions is used for levitation and rotation. This was commercialized by Tokyo Keiki Ltd. to be used for motion loggers for subways in Tokyo.

**Fig. 6:**
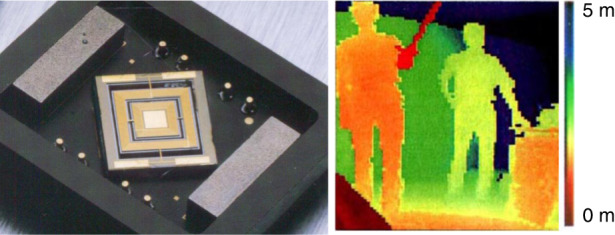
Electromagnetic two-axis MEMS scanner and a ranging image from the scanner^11^.

An electromagnetic two-axis optical scanner was developed and commercialized as a ranging imager by Nippon Signal Co. Ltd. as shown in Fig. 6^11^. Ranging images can be obtained using the time of flight of light, and the system has been used for platform doors in railway stations in Tokyo. Such systems are expected to be used in LiDAR (light detection and ranging) for future autonomous cars.

### Heterogeneous integration (MEMS on LSI)

Heterogeneous integration of MEMS on LSI was enabled by stacking the MEMS on the LSI^12^. This technology can be used to develop sophisticated MEMS systems. MEMS are fabricated on a carrier wafer to avoid damaging the LSI during MEMS fabrication. The MEMS wafer is bonded to the LSI wafer with adhesive resin or by bumps. The carrier wafer is removed, leaving the MEMS on the LSI wafer.

**Fig. 7:**
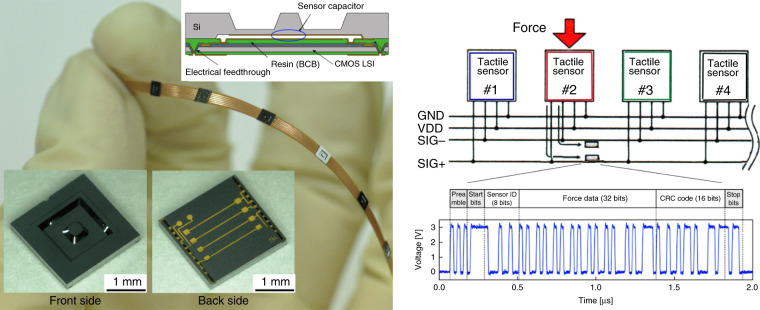
Tactile sensor network^14^.

Distributed tactile sensors (tactile sensor networks) are required on the skin of nursing care robots to ensure collision safety. The first tactile sensor network in our laboratory was developed in 1990 using IC fabricated in our laboratory^13^. However, it was a polling type and hence not real-time sensing because of the limited capacity of integration (1,000 transistors on a chip) in our laboratory. Since this, we gave up fabricating ICs in our laboratory and ordered LSI wafers from the foundry. The wafer is shared with noncompeting companies to reduce the cost. The tactile sensor with its structure and an example of its packet communication are shown in Fig. 7^14^. The MEMS wafer for capacitive force sensing is bonded with adhesive resin to the LSI wafer, which has a TSV (through-silicon via). Diced chips are connected to a flexible cable that has common signal bus and power supply lines. Packet signals for asynchronous communication use the common bus to enable real-time tactile sensing with a 45 MHz clock signal.

**Fig. 8:**
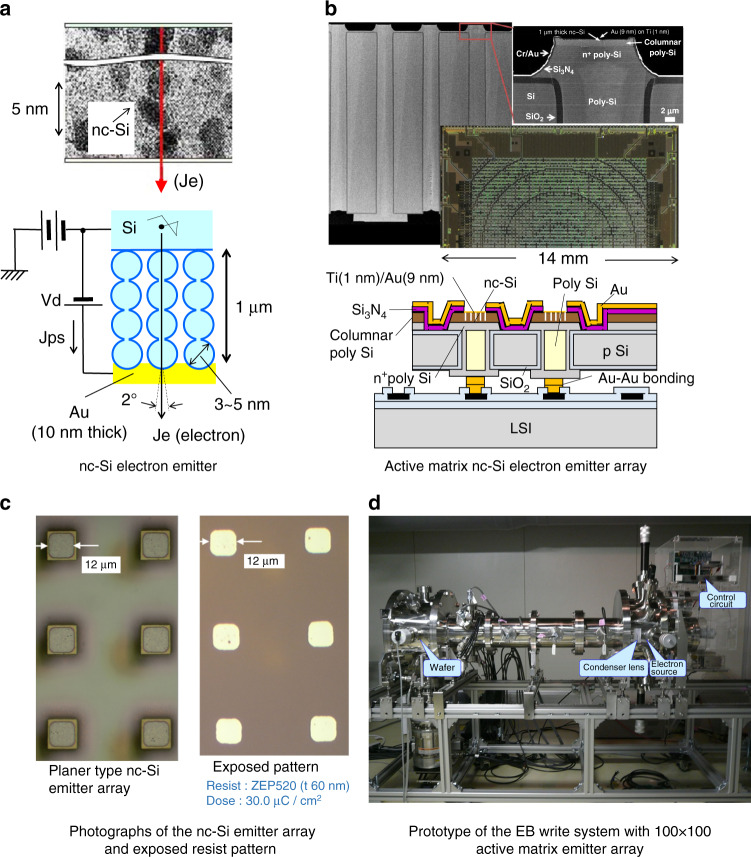
Massive parallel electron beam write system using a nc-Si electron emitter array^15^.

Digital fabrication of LSI by mask-less lithography is expected for cost-effective small-volume production and efficient development. MPEBW (massive parallel electron beam write) systems, which have a 100 × 100 active matrix electron emitter array, have been developed (Fig. 8)^15,16^. The electron emitter is made of nc-Si (nanocrystalline silicon). nc-Si consists of cascading tunnel junctions, and accelerated ballistic electrons are emitted through a thin (10 nm thick) Au layer by applying a low voltage (10 V) (Fig. 8a). The active matrix electron emitter and its structure are shown in Fig. 8b. Photographs of the nc-Si emitter array and the exposed resist pattern by 1:1 projection are shown in Fig. 8c. Figure 8d is a photograph of the prototype EB write system.

Heterogeneous integration by wafer-level transfer was applied for the PZT MEMS switch on LSI^17^, the BDD (boron-doped diamond) electrode array on LSI for electrochemical detection of biochemical substances^18^, and the FBAR (film bulk acoustic resonator) on LSI^19^.

**Fig. 9:**
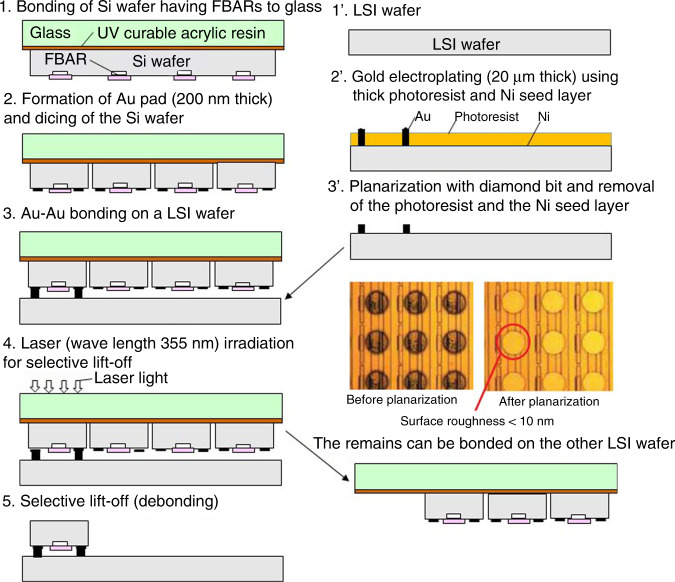
Fabrication process of multi FBAR on LSI by selective transfer^20^.

**Fig. 10:**
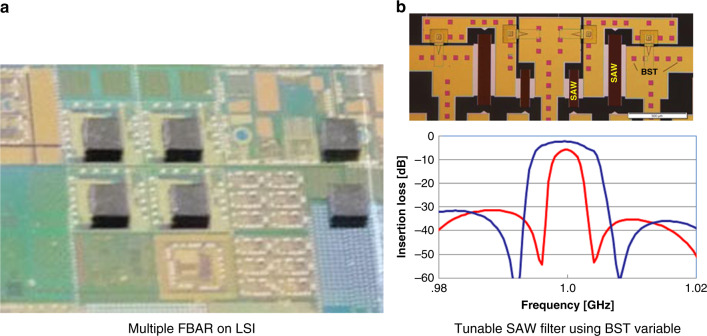
Multiple FBAR on LSI^20^ and tunable SAW filter using variable capacitors^21^.

The other heterogeneous integration method is chip-level selective transfer. This method is required when the size of the MEMS chip is different from that of the LSI chip. The selective transfer process by laser lift-off (debonding) is used as follows. Fig. 9 shows the fabrication process of multi FBAR on LSI^20^. A Si wafer on which FBARs are fabricated is bonded to a glass carrier wafer using UV curable acrylic resin (1 in the figure). Au pads are formed, and the Si wafer is diced (2 in the figure). Gold pads on an LSI wafer are fabricated by electroplating and planarization, as shown in 1’, 2’, and 3’ in Fig. 9. The FBAR wafer is aligned with the LSI wafer, and these wafers are pressed for Au−Au bonding (3 in the figure). Selective transfer by laser lift-off (debonding) is made by irradiating the interfacial acrylic resin using a Nd:YVO_4_ third harmonic laser (λ = 355 nm) through a glass carrier wafer (4 in the figure). The acrylic resin is carbonized to lose adhesion, and the FBAR chip is transferred to the surface of the LSI wafer (5 in the figure). The FBAR chips remaining on the glass carrier wafer can be transferred to another LSI wafer. In the opposite way, FBAR chips from different carrier wafers can be transferred on the same chip to the LSI wafer. Selective transfer technology was applied to fabricate multiple FBARs on LSI, as shown in Fig. 10a.

Chip-level selective transfer was also applied to fabricate a band-width tunable surface acoustic wave (SAW) filter, as shown in Fig. 10b^21^. BST (barium strontium titanate) variable capacitors are bonded selectively to the SAW filter chip.

### Common use facility and knowledge

**Fig. 11:**
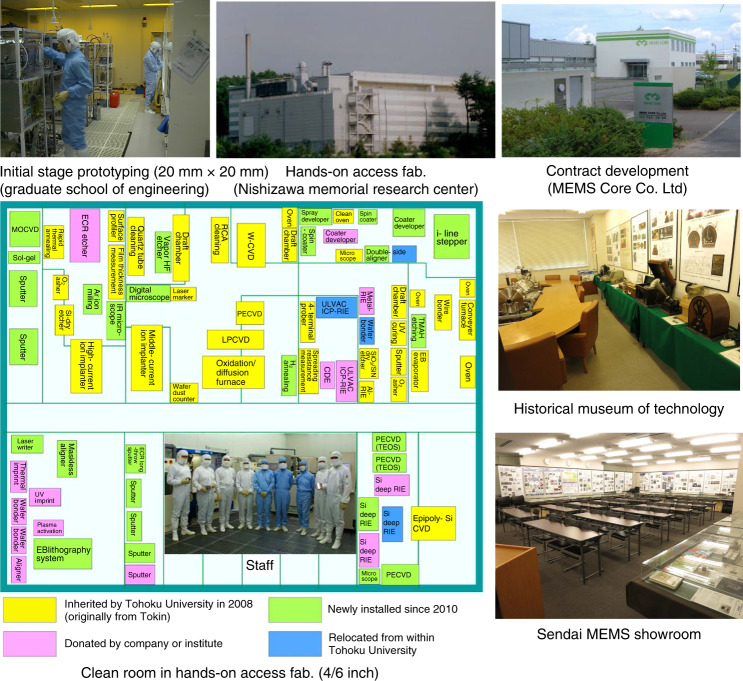
Layout of the clean room for the hands-on-access fabrication facility and photographs of other facilities available for open collaboration for MEMS development.

**Fig. 12:**
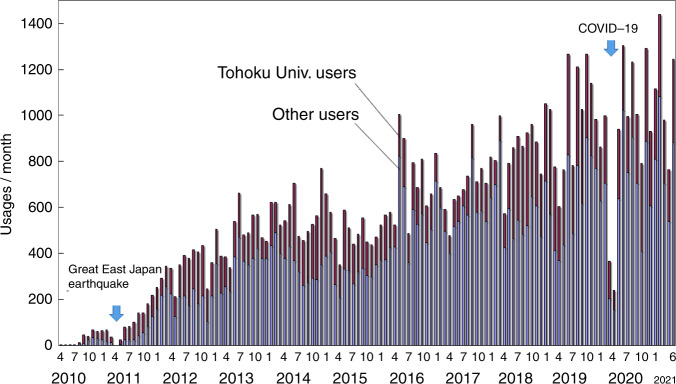
Monthly users of the hands-on-access fabrication facility.

MEMS are versatile and made in small volumes in many cases; hence, their development plays an important role. Various equipment with utilization factors that are not always high can be used. Thus, the common use of a facility for prototyping is effective for MEMS development. The hands-on-access fabrication facility (http://www.mu-sic.tohoku.ac.jp/coin_e/index.html), which was established in 2010, is open to every engineer working in this field^22^. The layout of the clean room of the facility is shown in Fig. 11. The facility is located in the Nishizawa Memorial Research Center at Tohoku University. Over 280 companies have dispatched their engineers to the hands-on-access fabrication facility. The facility has been managed by Prof. Kentaro Totsu. User engineers operate the process equipment by themselves with the support of technical staff. The available process equipment has been used previously in semiconductor factories. Simple old equipment can be maintained by technical staff and operated by engineers dispatched from companies. Engineers can gain experience with all of the processing steps and fabricate MEMS devices on 4- and 6-inch wafers. Product fabrication by company users has been allowed since 2013. Usages per month have increased over time, as shown in Fig. 12. The user fee in 2019 was approximately JPY 200 M, which is approximately 75% of the annual expenditure.

In addition to the hands-on-access fabrication facility, there are other facilities, as shown in Fig. 11. The initial stage prototyping facility, which is based on the made-in–house process equipment for a 20 mm square wafer, is located in the Graduate School of Engineering at Tohoku University. This is mainly operated by students and has flexibility for MEMS prototyping.

MEMS Core Co. Ltd. (President Mr. K. Honma) (http://www.mems-core.com/en/index.html) is a contract development company for MEMS devices and processes. The process equipment is mainly second-hand. It is hard for such a contract development company to survive; however, this company has been in business since 2001 owing to minimized investment by using the hands-on-access fabrication facility at Tohoku University.

Versatile knowledge is important for MEMS development. To provide efficient access to the knowledge, more than 1,000 files of MEMS technical papers can be found using keywords in Excel files. Furthermore, the MEMS book “3D and circuit integration of MEMS” was published and is available^23^.

There are exhibition rooms for viewing MEMS devices (http://www.mu-sic.tohoku.ac.jp/nishizawa_E/index.html), such as the “Sendai MEMS showroom” and the “Historical museum of technology” in the Nishizawa Memorial Research Center.

## Discussion

My MEMS research at Tohoku University for approximately half a century is reviewed in this article. Open collaboration is important, especially between universities and industry, because development is in many cases the bottleneck for MEMS commercialization. Facilities for MEMS prototyping should be flexible and accessible to allow engineers to gain experience. Made-in-house process equipment for initial prototyping and hands-on-access fabrication facilities for commercialization have been effective for use as common facilities.

In my experience with the supporting industry, I have learned that diversity is very important for the MEMS business^24^. In the case of LSI, diversity can be determined by programming, such as FPGA (field-programmable gate array). On the other hand, MEMS diversity requires the individual development of devices using fabrication facilities. Large volume MEMS and standard process MEMS can work like LSI, but investment in a facility is not cost-effective in many cases of small volume and nonstandard MEMS. Various approaches have been proposed for nonstandard and small-volume MEMS^25^. There are ways to solve this problem, and I think we should try to find additional approaches to lower the barriers and expand the MEMS business to diverse fields.
